# Some methodological issues in the development of public health recommendations from field trials of new malaria vector control interventions

**DOI:** 10.1186/1475-2875-11-S1-P134

**Published:** 2012-11-09

**Authors:** Jo Lines

**Affiliations:** 1 London School of Hygiene & Tropical Medicine, Keppel St, London WC1E 7HT, UK

## 

In this paper, I consider some technical aspects of the process by which public health recommendations are inferred from the results of epidemiological field trials and other research on new vector control products and technologies. I argue that the conventional Cochrane Collaboration methods for summarising clinical trials may need to be modified and extended in order to be valid when applied to vector control.

With medical interventions, for which the Cochrane methods were originally designed, the primary mode of action of the intervention works through biological processes within an individual, and the individual person is a natural experimental unit. This makes it easier to assume that a series of trials from different locations are more or less replicates of each other: they are asking more or less the same question, and in the absence of bias and sampling error they should produce more or less the same answer. In the case of vector control, on the other hand, the intervention has its most direct effects on mosquitoes, and the causal chain that leads to health benefits mostly occurs outside people, in the external environment. For this reason, in order to summarise a series of vector control trials, it is necessary to standardise not only the human populations, the interventions and the observed outcomes, but also the local vector populations and the broader ecological characteristics of the settings where the trials were carried out. Only with this additional matching is it valid to assume that vector control trials carried out in different settings are asking the broadly same question about the same intervention.

The same applies to the process of generalising from a set of field trials in specific settings to broader public health recommendations. We know from first principles that a vector control intervention that is effective in one setting may not be so in another. Moreover, the field trials of a new intervention can include only a small subset of the likely range of settings where the intervention might eventually be used. Thus we need systematic methods for asking not just “whether” an intervention will be effective, but also “where and when” it will be effective. In the past, malariologists have used “eco-epidemiological stratification” to deal with this problem: this approach now needs to be made more systematic and integrated.

Particular attention therefore needs to be given to the “epidemiological mode of action” of entomological interventions. This is the causal chain by which effects on mosquitoes lead to reduced infective biting for at least some people (e.g. “personal protection” and “the mass effect” caused by ITNs). This useful new concept, which has emerged from current discussions within the vector control product development community, appears to play a central role in the interpretation of evidence from vector control field trials for public health purposes.

